# Antiepileptic Efficacy and Network Connectivity Modulation of Repetitive Transcranial Magnetic Stimulation by Vertex Suppression

**DOI:** 10.3389/fnhum.2021.667619

**Published:** 2021-05-13

**Authors:** Cong Fu, Aikedan Aisikaer, Zhijuan Chen, Qing Yu, Jianzhong Yin, Weidong Yang

**Affiliations:** ^1^Department of Neurosurgery, Tianjin Medical University General Hospital, Tianjin Medical University, Tianjin, China; ^2^Department of Radiology, Tianjin First Central Hospital, Tianjin Medical University, Tianjin, China; ^3^Department of Neurology, Tianjin Medical University General Hospital, Tianjin Medical University, Tianjin, China

**Keywords:** rTMS, refractory epilepsy, BOLD fMRI, functional network connectivity, sensorimotor network, default mode network

## Abstract

A core feature of drug-resistant epilepsy is hyperexcitability in the motor cortex, and low-frequency repetitive transcranial magnetic stimulation (rTMS) is a suitable treatment for seizures. However, the antiepileptic effect causing network reorganization has rarely been studied. Here, we assessed the impact of rTMS on functional network connectivity (FNC) in resting functional networks (RSNs) and their relation to treatment response. Fourteen patients with medically intractable epilepsy received inhibitive rTMS with a figure-of-eight coil over the vertex for 10 days spread across two weeks. We designed a 6-week follow-up phase divided into four time points to investigate FNC and rTMS-induced timing-dependent plasticity, such as seizure frequency and abnormal interictal discharges on electroencephalography (EEG). For psychiatric comorbidities, the Hamilton Depression Scale (HAM-D) and the Hamilton Anxiety Scale (HAM-A) were applied to measure depression and anxiety before and after rTMS. FNC was also compared to that of a cohort of 17 healthy control subjects. The after-effects of rTMS included all subjects that achieved the significant decrease rate of more than 50% in interictal epileptiform discharges and seizure frequency, 12 (14) patients with the reduction rate above 50% compared to the baseline, as well as emotional improvements in depression and anxiety (*p* < 0.05). In the analysis of RSNs, we found a higher synchronization between the sensorimotor network (SMN) and posterior default-mode network (pDMN) in epileptic patients than in healthy controls. In contrast to pre-rTMS, the results demonstrated a weaker FNC between the anterior DMN (aDMN) and SMN after rTMS, while the FNC between the aDMN and dorsal attention network (DAN) was greater (*p* < 0.05, FDR corrected). Importantly, the depressive score was anticorrelated with the FNC of the aDMN-SMN (*r* = −0.67, *p* = 0.0022), which was markedly different in the good and bad response groups treated with rTMS (*p* = 0.0115). Based on the vertex suppression by rTMS, it is possible to achieve temporary clinical efficacy by modulating network reorganization in the DMN and SMN for patients with refractory epilepsy.

## Introduction

Transcranial magnetic stimulation (TMS) is a safe and well-tolerated noninvasive focal cortical stimulation technique (Pereira et al., 2016; Tsuboyama et al., 2020) based on the theory of Faraday electromagnetic induction (Barker, 1999). Through a series of magnetic pulses acting on the cerebral cortex, neural activity is inhibited under low-frequency stimulation. Because of its hypothesized mechanism of action with enhancement of GABAergic activity (Pascual-Leone et al., 1994) and a decrease in synaptic transmission (Chen et al., 1997; Ye and Kaszuba, 2019), low-frequency repetitive TMS (rTMS) might be ideally suited to epilepsy pathophysiology. An important characteristic of the epileptic brain is cortical hyperexcitability due to disruption of brain networks (Cantello et al., 2000) and abnormal imbalance of cortical excitability and sensitivity (Tombini et al., 2013), which supports excitation-inhibiting rTMS as a potential therapeutic approach (Badawy et al., 2012; Kramer and Cash, 2012).

For refractory patients with multiple foci or diffuse epileptiform foci, the vertex region (in SMN) was used as the untargeted rTMS site, which could be located in Cz according to the 10-20 electroencephalography (EEG) system (Tergau et al., 1999). In a randomized, double-blind, crossover design study for 43 new cortex focal epilepsy patients selected for 26 weeks trial, TMS therapeutic targeted a vertex area and results showed that the true stimulus group compared with sham stimulus clinical performance with no significant difference, but the EEG detected that a third of the patients gained improvement on epileptiform discharge (Cantello et al., 2007). Kinoshita et al. (2005) found that compared with vertex stimulation of simple focal seizures, complex focal seizures were more obvious in the improvement of seizure, which explained that the vertex inhibitory stimulus did not prevent the occurrence of epileptic activity but prevented the proliferation of abnormal brain activity (Kinoshita et al., 2005). Therefore, as observed in the beneficial result from patients with diffuse or multiple foci epilepsy, it might be that the vertex caused the network effect, making the whole epilepsy network excitatory downgrade (Tergau et al., 2003; Joo et al., 2007). A recent study from Yun and colleagues suggested that SMN may have implications for intervention in generalized epilepsy due to SMN in the subcortical-cortical circuit (Qin et al., 2020). The above studies provide theoretical and practical support for the regulation of excitation-inhibitory rTMS, and the vertex areas (in SMN) are regarded as an untargeted site to modulate the functional network for an antiepileptic effect.

Functional network connectivity (FNC) is a technique based on resting-state fMRI that identifies connectivity between contributed resting-state networks (RSNs) based on correlations over time in the blood oxygenation level-dependent (BOLD) signal. TMS has been combined extensively with FNC to identify abnormalities in brain connectivity in different neurologic and psychiatric diseases (Fox et al., 2012a, b; Chou et al., 2015). However, there are few studies about the rTMS vertex-suppressive effect causing FNC reorganization in refractory epilepsy.

Here, the first aim of the study was to examine the effect of vertex desynchronization by rTMS in terms of seizure frequency, abnormal EEG discharges, and depressive and anxious scores. The second aim was to use FNC analysis to identify network connectivity reorganization that was modulated by the rTMS intervention and assess whether rTMS-induced functional connectivity modulation was associated with changes in clinical symptoms.

## Materials and Methods

### Subjects

Seventeen right-handed drug-resistant epilepsy patients who were not eligible for surgical treatment were recruited from the outpatient and inpatient department of neurology and neurosurgery. Drug resistance was defined in agreement with commonly accepted criteria (Schmidt and Löscher, 2005; Kwan et al., 2010). According to the criteria of the International League Against Epilepsy (ILAE) Board, epileptic syndrome and seizure type were definitively diagnosed (ILAE, 1989). We excluded three patients with serious structural abnormalities. Eventually, 14 patients were included in our baseline assessment {six females, age 26.72 (8.13) years [mean (standard deviation)]}. During the experiment, there were four dropout patients due to individual reasons. In the end, 10 patients finished the whole experiment (see Supplementary Figure 1). The diagnostic and clinical characteristics of the patient group are described in Table 1. Additionally, 17 age- and sex-matched healthy adults [nine females, age 25.29 (1.86) years] were recruited from the local community. All subjects were eligible for MRI based on standard MRI safety screening; all patients passed the TMS adult safety–screening questionnaire (TASS; Keel et al., 2001; except for the epilepsy-related questions). All subjects gave written and informed consent.

**Table 1:** Patients’ clinical characteristics.

Clinical characteristic	Patients (No.)
Seizures	
Motor seizures	14
Focal	2
Generalized	12
Epilepsy	
Focal	2
Multifocal	2
Generalized	12
Multifocal	12
Comorbidities	
Depression	
Intellectual dysfunction	3
Motor deficits	
Gait	2
Movement disorders	3

### Research Protocol

**Figure 1:**
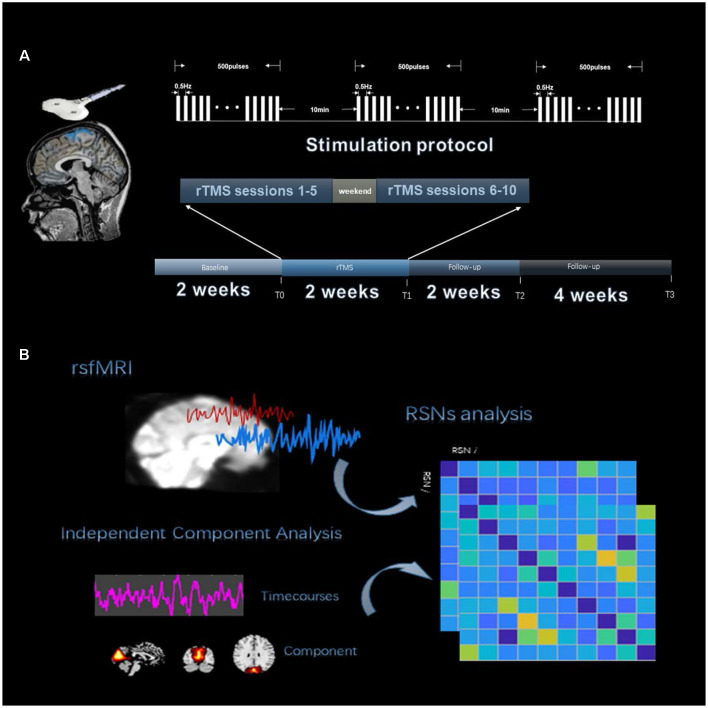
Experiment procedure. **(A)** The four time points were designed into the timeline of the study, which were T0, T1, T2, and T3. Repetitive transcranial magnetic stimulation (rTMS) 10 sessions with the stimulation protocol are shown above the timeline. **(B)** The analysis process of functional network connectivity (FNC) divided into three steps, including fMRI data preprocessing, resting-state network (RSN) identification by independent component analysis (ICA) and extracting RSN time courses to calculate Pearson’s correlation.

There were 10 weeks for patients to participate (see Figure 1A). We performed rTMS in 10 sessions over 2 weeks, one session per day for five consecutive weekdays each week. The baseline assessment T0 occurred 2 weeks prior to the stimulation session, and there were 10 daily stimulation sessions. Post-rTMS assessment, T1, T2, and T3 occurred on the first day, the end of the 2nd week, and the 6th week after the final stimulation session. At each time point, we monitored patients’ seizure frequency. Imaging data were collected, including structural MRI and blood oxygen level-dependent (BOLD) fMRI, for each time point (T0–T3) using the parameters indicated below. Following MRI scanning, subjects underwent video electroencephalography (VEEG) and emotional assessment, as described below. Each healthy subject participated in imaging data collection, including structural MRI and BOLD fMRI.

### Repetitive Transcranial Magnetic Stimulation (rTMS)

rTMS was applied to the stimulation location using a CCY-IA TMS instrument (YIRUIDE Limited, China). A 70 mm figure-of-eight coil was used. For the stimulation condition, rTMS was applied at a 100% motor threshold, which was necessary to generate a visible contraction of the right thumb (abductor pollicis brevis) for 5 out of 10 consecutive pulses, to the stimulation site for 1,500 pulses of 0.5 Hz pulses for every 500 pulses followed by 10 min of interval stimulation (see Figure 1A). A coil was positioned over the motor cortex (at Cz), which was targeted using a 10-20 EEG standard location system. Each patient received rTMS at the same time every afternoon (2:00 PM). During the study period, antiepileptic medications were kept constant.

### Clinical Evaluation and Rating Scales

At the T0–T3 phases, patients underwent medical and neuropsychological examinations. The mean number of weekly seizures (MNWS) could represent the main indicator of the antiepileptic effect on rTMS. All patients and their families documented every seizure before and after the low-frequency rTMS treatment. To compare the MNWS of three phases immediately subsequent to the rTMS treatment, we performed a one-way analysis of variance (ANOVA) and time (T0, T1, T2 and T3) as a repeated factor. Similarly, EEG comparisons were performed for the absolute number of interictal epileptiform discharges (IEDs) within the second 30 min of 1.5 h of detection (Varrasi et al., 2004). The Hamilton Depression Scale (HAM-D) and Hamilton Anxiety Scale (HAM-A) were used by a skilled psychologist (M.M.) to measure the patients’ emotions, and the mean value of the total score was recorded as a statistical indicator. The significance level was set to *p* < 0.05.

### Imaging Protocol

All MRI scanning data were obtained on a 3-tesla MRI scanner (Siemens Prisma). High-resolution T1-weighted data images were acquired using a magnetization–prepared rapid gradient–echo (MPRAGE) sequence (repetition time (TR) = 1,550 ms, echo time (TE) = 2.98 ms, field of view (FOV) = 256 mm ×256 mm, slice thickness 1.00 mm, 176 volumes). Resting-state functional BOLD (Bonilha et al., 2010) data images were acquired using an echo planar imaging (Wiebe et al., 2001) sequence (TR = 2,000 ms, TE = 30.00 ms, flip angle 40°, FOV = 220 mm ×220 mm, slice thickness 2.5 mm, 380 volumes). The patients were asked to not move and to stay with their eyes closed and resting. Headphones and cushions were used to reduce noise interference and prevent excessive head movement.

### fMRI Data Analysis

Preprocessing of the data was performed according to graph-theoretical network analysis. The toolkit (GRETNA^1^) fMRI preprocessing pipeline included slice-timing correction, head motion correction, spatial normalization, and smoothing. The following denoising steps were performed with the unsmoothed images (Wang et al., 2015): detrending, temporal bandpass filtering, and removal of nuisance signals by regression on head motion effect, white matter, and cerebrospinal fluid signals, and an indispensable “scrubbing” procedure. Finally, no patient had fewer than 300 volumes. Additional preprocessing information is described in the Supplementary Material.

**Figure 2:**
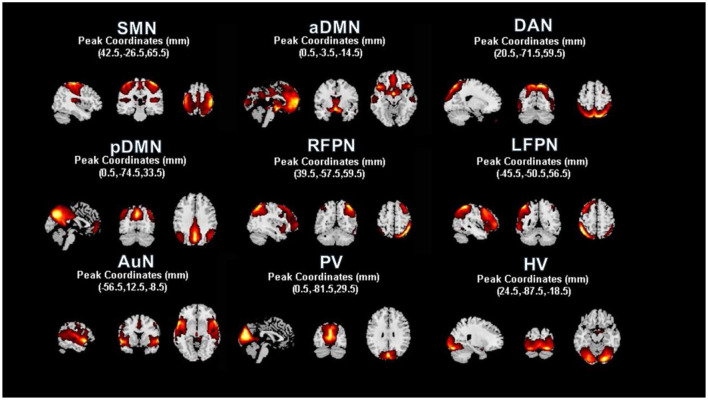
Resting state functional networks identified by ICA. We identified nine meaningful RSNs and extracted the corresponding mean time courses. In each RSN, peak coordinates in Montreal Neurological Institute (MNI) space helped us to verify the location of these nine built networks. A one-sample *t*-test was used to find the significant voxels within their networks (*p* < 0.01, voxel correlation *via* FDR).

We used the group independent component analysis (GICA) method to extract the spatial components of nine defined RSNs as shown in Figure 1B. The GICA of the fMRI toolbox^2^ was used for each group of participants for their respective group spatial ICA, and more details are provided in the Supplementary Material. Spatial components of nine cortical RSNs were gathered across each group by one-sample *t*-tests, including Attention Network (AN), Anterior Default Modal Network (DMN), Posterior Default Modal Network (DMN), Sensorimotor Network (SMN), Right Frontoparietal Network (RFPN), Left Frontoparietal Network (LFPN), Prim-visual Network (PV), High-visual Network (HV) and Auditory Network (AuN), as shown in Figure 2 (*p* < 0.01 for multiple comparisons corrected *via* false discovery rate). The abbreviation rules of nine RSNs were in terms of the Stanford brain functional template^3^ and prior studies (Corbetta and Shulman, 2002). Finally, 36 statistical maps were converted to 36 binary masks and were shown with BrainNet Viewer (Xia et al., 2013) in MATLAB.

The corresponding mean time series of the 36 RSNs were extracted with REST software (Song et al., 2011), and FNCs in the epilepsy groups (T0, T1, T2, and T3) and healthy control group were calculated. We obtained 9 × 9 FNC mean matrices of all the subjects and performed Fisher’s *r* to *z* transformation. We compared FNC results in four time windows: “baseline” before treatment (T0) and the two to four phases of “posttreatment follow-up” after treatment (T1–T3). Four pairwise comparisons were performed using two-sample *t-tests* between patients and HCs (*p* < 0.05, FDR corrected), with age, sex, and head motion (mean FD) as nuisance covariates. The significance level was set at *p* < 0.05 and corrected for multiple comparisons using the false discovery rate (FDR). The three follow-up groups were compared to the pretreatment groups using two-sample *t-tests* (*p* < 0.05, FDR corrected).

## Results

### Subject Characterization and Antiepileptic Effects of rTMS

Fourteen patients with refractory epilepsy were enrolled in this study, and all completed the 2-week course of rTMS over the vertex. There was no adverse effect on rTMS treatment reported from patients. Patients and healthy controls (*N* = 17) did not differ significantly in terms of age (*t* = 0.70, *p* = 0.49), sex (*Χ*^2^ = 0.31, *p* = 0.58) or handedness (Supplementary Table 1).

**Figure 3:**
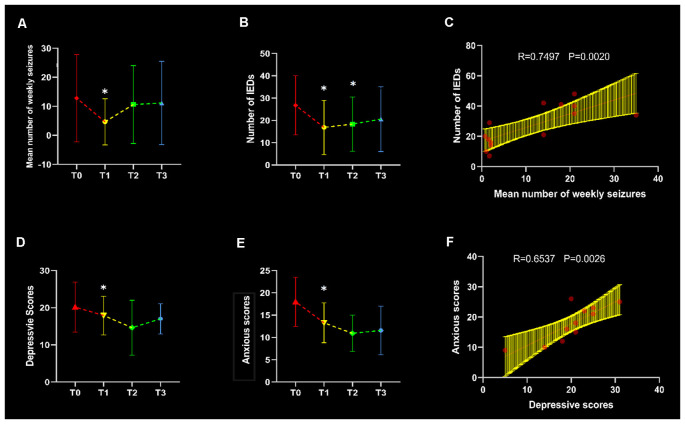
Clinical evaluation and correlation. Line graphs were plotted in terms of the comparisons between three follow-up time points and baseline (*p* < 0.05), with asterisks indicating significant changes. The mean number of weekly seizures (MNWS), interictal epileptiform discharges (IEDs), depressive scores and anxious scores in each time points were shown in panels **(A,B,D)** and **(E)** respectively. In the T0 baseline, IEDs showed an increased association with MNWS in **(C)**, and a positive correlation was found between depressive and anxious scores in **(F)**.

After rTMS treatment, the mean number of weekly seizure (MNWS) across the patients for T1, T2, and T3 was 4.67 ± 7.94, 10.61 ± 13.42, and 11.17 ± 14.30 (mean ± standard deviation). Compared with 12.82 ± 15.06 in T0, 12 (14) patients exhibited a decreased MNWS with a statistical significance, *F*_(1.11, 14.48)_ = 10.15, *p* = 0.01. After Bonferroni *post hoc* test, we found there was a noticeable reduction in T1 (*p* = 0.03) while no significant change was found in T2 (*p* = 0.05) and T3 (*p* = 0.10) as shown in Figure 3A.

All patients showed a decreased IEDs and an ANOVA of the IEDs yielded a significant main effect of time points, *F*_(2.04, 26.51)_ = 8.81, *p* < 0.01. Through the pairwise comparison, results revealed that T1 (*p* = 0.04) and T2 (*p* < 0.01) showed a significant decrease after rTMS intervention but no discrepant change was found in T3 (*p* = 0.08) in Figure 3B. In addition, there was a significant correlation between MWSF and IEDs (*r* = 0.7494, *p* = 0.002) at baseline in Figure 3C, which meant the consistency of the clinical indicators.

Through the repetitive measurement ANOVA, the result produced markable differences in HAM-D and HAM-A scores, *F*_(3,18)_ = 5.216, *p* = 0.009, and *F*_(3, 18)_ = 8.302, *p* = 0.001. After the *post hoc* test, we found that patients’ symptoms improved on the HAM-D at T1 compared to baseline (*p* = 0.022) in Figure 3D. Moreover, anxiety assessment found decreased symptoms in T1 (*p* = 0.015) in Figure 3E. And there was a significant correlation between depressive and anxious scores in T0 (*r* = 0.6527, *p* = 0.0026) in Figure 3F.

### Functional Network Connectivity

**Figure 4:**
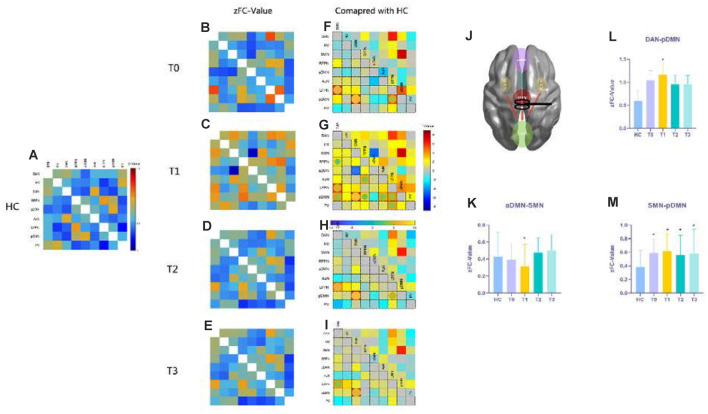
Functional network connectivity (FNC) results compared with those of HCs. Panels **(A–E)** show the resting functional network results with FNC values that underwent Fisher’s *r* to *z* transformation, referring to the color bar in **(A)**; **(F–I)** the *t*-maps give the information about the contrast of FC compared to HCs at each time point, the significant differences emphasized with spheres and their colors mean different *t*-values (*p* < 0.05, FDR corrected), referring to the horizontal bar in **(G)**; panels **(K–M)** show the FNC *z*-values for SMN-aDMN, SMN-pDMN, and pDMN-DAN connections in HCs and patients at each time point with the asterisks indicating a significant difference compared with HCs (*p* < 0.05, FDR correlated). **(J)** A conceptional diagram with red lines representing hyperconnectivity and the blue line meaning hypoconnectivity after rTMS treatment. The nine functional networks are abbreviated in Supplementary Table 2. Abbreviations: HCs, healthy controls; SMN, sensorimotor network; aDMN, anterior default-mode network; pDMN, posterior default-mode network; DAN, dorsal attention network.

Functional network connectivity in the epilepsy groups (T0, T1, T2, and T3) and healthy control group were produced as shown in Figures 3A–E. We made comparisons between healthy controls and patient groups at each time point (T0–T3) with the covariates of age, sex, and head movement. The FNC results showed significant differences at the first follow-up time point (T1) shown in Figures 4G and 4J while the baseline T0 in 4F and subsequent follow-ups (T2 in Figure 4H and T3 in Figure 4I) were almost the same as HCs, with the exception of the posterior default-mode network (pDMN)–SMN connectivity (T2 *t* = 6.19, *p_FDR_* < 0.001; T3 *t* = 6.10, *p_FDR_* < 0.001) shown in Figure 4M. Importantly, we found that SMN–anterior DMN (aDMN) connectivity decreased after rTMS intervention at T1 (*t* = −4.85, *p_FDR_* < 0.001) shown in Figure 4K. With respect to the final follow-up, T3 returned back to the baseline level in T0.

**Figure 5:**
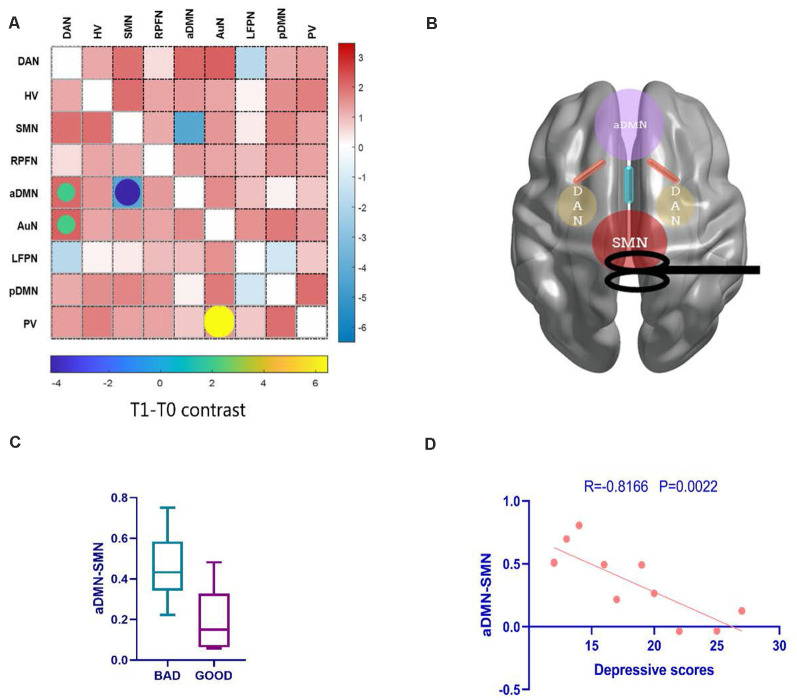
The alteration of FNC and their clinical correlations after rTMS. **(A)** Posttreatment FNC in T1 shows a decreasing change in internetwork connectivity between the SMN and aDMN, while the DAN-aDMN, DAN-AuN, and PV-AuN pairs demonstrated hyperconnectivity after rTMS. **(B)** A conceptional graph of the important and temporary changes in T1. **(C)** Comparing bad and good response groups in T1, a two-sample *t*-test was used to test the significant difference in FNC *z*-value of aDMN-SMN (*p* = 0.0115). **(D)** With the correlation analysis of the FNC in the aDMN-SMN and depressive scores, the results showed a more than moderate correlation (Pearson’s correlation *r* = −0.67, *p* = 0.0022). AuN, auditory network.

After 10 days rTMS treatment for the patient group, the aDMN showed temporarily lower connectivity with the SMN (*t* = −4.2446, *p_FDR_* < 0.001), and hyperconnectivity with the dorsal attention network (DAN; *t* = 2.0828, *p_FDR_* = 0.0472) appeared in T1 (Figures 5A and Figure 5B), which could not be found in further follow-ups of T2 and T3. Moreover, T2 and T3 demonstrated no significant alteration in contrast with T0 (*p_FDR_* > 0.05). Then, according to the improvement in clinical seizure frequency on T1, we divided the patients into two groups: bad and good response on rTMS. Patients who showed a better response to rTMS were statistically distinguishable from the *z*-value of aDMN-SMN by the two-sample *t*-test (*t* = 2.980, *p* = 0.0115). However, it was temporary because T2 and T3 were not found (Figure 5C). To test whether the *z*-value of aDMN-SMN in T1 was related to treatment response, we used Pearson’s correlation to find that the *z*-value of aDMN-SMN was anticorrelated with depression scores of T1 in Figure 5D (*r* = −0.67, *p* = 0.0022), which meant an improvement in depressive symptoms. Other clinical measures showed no significance with the FNC *z*-value of aDMN-SMN.

## Discussion

Epilepsy is an abnormal network disease and it is potential to use network modulation in order to an antiepileptic efficacy (Tecchio et al., 2018). Our study demonstrated the antiepileptic effect of low-frequency rTMS over the vertex (in SMN) in refractory epilepsy patients. Due to our strict work on patient filters, almost every patient had visible movement seizures during the ictal period and more than once a week. The positive outcome was achieved in the first follow-up but disappeared in the following observation periods. The results on the persistent period of rTMS after-effects are consistent with previous studies, which would prolong the antiepileptic efficacy for no more than 6–8 weeks posttreatment observation period (Theodore et al., 2002; Fregni et al., 2006; Sun et al., 2012), suggesting that a long-term after-effect are needed for sustainable treatment.

For FNC analysis, the present study investigated whether vertex-suppressive rTMS could modulate brain functional networks in patients with refractory epilepsy and whether modulated functional connectivity was associated with changes in clinical symptoms. To investigate RSN reorganization induced by the antiepileptic effect of rTMS on vertex suppression, we compared the changes in resting-state FNC before and after rTMS. Our findings suggest that a 10-session 0.5-Hz rTMS targeting the vertex may improve epileptic symptoms by modulating functional links connecting to the SMN, DMN, and DAN for patients with refractory epilepsy.

In contrast to healthy subjects, the SMN showed a higher FNC with the pDMN in patients with refractory epilepsy, and the posterior cingulate cortex (PCC) is a core region in the DMN that is associated with autobiographical, self, and social functions (Buckner et al., 2008). Previous studies proposed that the PCC was crucial for the motor circus, suggesting that the abnormal function of the PCC might impact the motor circus through projections from the PCC to the anterior thalamus (Yeterian and Pandya, 1988). And a recent study demonstrated that thalamic hyperexcitability contributed to the cortical maintenance of epileptic susceptibility in juvenile myoclonic epilepsy (Assenza et al., 2020a). Because there are direct projections from the thalamus to the PCC, abnormal IEDs from the thalamus lead to precuneus/PCC abnormalities (Avoli et al., 2001; Gotman et al., 2005). The abnormal functional activity in the PCC was associated with impairment of awareness in the ictal period (Archer et al., 2003; Jia et al., 2018), which might partly explain consequent attention disorders during the interictal period (Brandt, 1984; Lui et al., 2008). As most patients in our study had generalized motor seizures accompanied by consciousness disorders, the enhanced coupling between the SMN and PCC (in the pDMN) might suggest an overexciting motor circus in refractory epilepsy. However, vertex-suppressive rTMS did not show changes in SMN-pDMN connectivity in the treatment groups.

Due to its special location in the distributed functional network, as in previous studies, we use the vertex (in SMN) as the stimulus point (Tergau et al., 2003; Cantello et al., 2007). The sensorimotor network, as demonstrated in a wealth of studies, is partially integrated into a multimodal network associated with motor systems and cognitive hubs (Sepulcre et al., 2012). After rTMS intervention, RSN temporary reorganization appeared among the aDMN, SMN, and DAN. We used inhibitive rTMS to lower neural activity in the SMN, while the aDMN showed lower synchrony with the SMN and higher synchrony with the pDMN. The DMN showed an active state in response to rTMS that was considered to be involved in a high degree of neuroplasticity (Raichle et al., 2001; Fjell et al., 2014). In the correlation analysis, we found that SMN-aDMN connectivity was anticorrelated with depressive scores and related to seizure improvement. Our results are consistent with previous fMRI studies, suggesting that medial prefrontal cortex (in the aDMN) disruption has been implicated in changes in emotional impairments (Satpute and Lindquist, 2019). For treat-resistant patients, a recent fMRI study of the DMN has found that internetwork connectivity of the DMN presented an anticorrelation with the duration of epilepsy, suggesting that DMN connectivity might be a predictor of the antiepileptic effect (Yang et al., 2021).

Several limitations should be acknowledged while interpreting our results. On the one hand, sham stimulation groups are omitted, as the stimulator’s factors were overcome by our manipulations, such as choosing noiseless equipment and no extra physical contact with patients. Moreover, we included IEDs as objective outcome measures that could reflect a more impersonal evaluation. On the other hand, we hope to add subcortical nuclear analysis, such as analysis of the thalamus and its posterior movement circuit of the basal ganglia, in our further studies. As mentioned above, the thalamus plays a hub role related to the pathological manifestation in epilepsy (Bestmann et al., 2004; Jobst and Cascino, 2017; Assenza et al., 2020a,b). Basal ganglia involved in movement circus discussion may help to better explain our results with respect to the interaction between the pDMN and SMN.

## Data Availability Statement

The original contributions presented in the study are included in the article/Supplementary Material, further inquiries can be directed to the corresponding author/s.

## Ethics Statement

The study protocol was approved by the Tianjin Medical University Institutional Review Board. The patients/participants provided their written informed consent to participate in this study.

## Author Contributions

JY and CF: conceptualization. CF: methodology and writing—original draft preparation. ZC, AA, and QY: validation. AA and QY: resources. AA and ZC: data curation. JY and WY: writing—review and editing. WY and JY: supervision. WY: funding acquisition. All authors contributed to the article and approved the submitted version.

## Conflict of Interest

The authors declare that the research was conducted in the absence of any commercial or financial relationships that could be construed as a potential conflict of interest.

## Footnotes

^1^http://www.nitrc.org/projects/gretna/

^2^https://www.nitrc.org/projects/gift

^3^http://findlab.stanford.edu/functional_ROIs.html
