# Supplementary material for: Antiepileptic Efficacy and Network Connectivity Modulation of Repetitive Transcranial Magnetic Stimulation by Vertex Suppression
Source: Front Hum Neurosci. 2021 May 13;15:667619. doi: 10.3389/fnhum.2021.667619 (PMC8155627; doi:10.3389/fnhum.2021.667619)
Supplement: Supplementary file 1 [file Table_1.DOCX]

Supplementary Material

# Supplementary Data

# Methods details

# Preprocess

The first 10 volumes were removed, and then slice-timing correction and head motion correction were performed. The data from patients with head motion exceeding 2 mm or head rotations greater than 2° were excluded from further calculations, while head motion in controls was limited 1 mm or 1°. The motion-corrected functional images were normalized to the standard Montreal Neurological Institute (MNI) space by applying an EPI template, which led to our data showing a better match with the EPI template (Calhoun et al., 2017). Subsequently, to avoid mixing white matter and grey matter signals, the normalized images were spatially smoothed using a 4-mm full-width half-maximum Gaussian kernel. The acquired smoothed data were utilized in independent component analysis (ICA). The following denoising steps were performed with the unsmoothed images (Wang et al., 2015): (1) removing the linear trends of time courses; (2) bandpass filtration (0.01–0.08 Hz) to minimize the influence of low-frequency drifts and high-frequency physiological noise; (3) linear regressing out the confounding signals that were unlikely to reflect neural activity, including the head motion effect (Friston 24 parameter) (Friston, Williams, Howard, Frackowiak, & Turner, 1996), white matter and cerebrospinal fluid signals; and (4) an indispensable ‘scrubbing’ procedure(Power, Barnes, Snyder, Schlaggar, & Petersen, 2012). Concretely, in terms of the criteria of framewise displacement (FD) above 0.5 mm, functional imaging data presenting sudden head motion were discarded, together with one volume before and two volumes after the bad volume(Power, Schlaggar, & Petersen, 2015).

**ICA**

The data were decomposed into 20 components that were estimated by GIFT, including data reduction by PCA, ICA separation, and back-reconstruction. Two-step PCA was used for data reduction. The maximum likelihood algorithm was used for group-level spatial ICA. A regular algorithm was used for stability analysis, and GICA was used for back-reconstruction. Each subject obtained a spatial component and the corresponding time-series component, and correlation coefficients were converted to a normal distribution by Fisher’s r-to-z transformation. For each component selection, we obeyed the selection criterion. In particular, ICA selection was independently completed by 2 senior neuroimaging physicians (J.Y.andA.A.) and referred to corresponding templates(<http://findlab.stanford.edu/functional_ROIs.html>).

# Supplementary Tables

**Supplement Table 1**

|  | HC | Patients | *p* value* |
| --- | --- | --- | --- |
| Sex(female/male) | 9/8 | 6/8 | 0.58 |
| Age [Mean (SD)] | 25.29 (1.86) | 26.72(8.13) | 0.49 |
| Handedness(left/right) | 0(17) | 0(14) | 1.00 |

*student *t*-test for the continuous variables and Fisher exact’s test for the categorical variables.

SD – standard deviation.

**Supplement Table 2**

| Networks | Abbreviation |
| --- | --- |
| Attention Network | AN |
| Anterior Default Modal Network | aDMN |
| Posterior Default Modal Network | pDMN |
| Sensorimotor Network | SMN |
| Right Frontoparietal Network | RFPN |
| Left Frontoparietal Network | LFPN |
| Prim-visual Network | PV |
| High-visual Network | HV |
| Auditory Network | AuN |

## Supplementary Figures

**
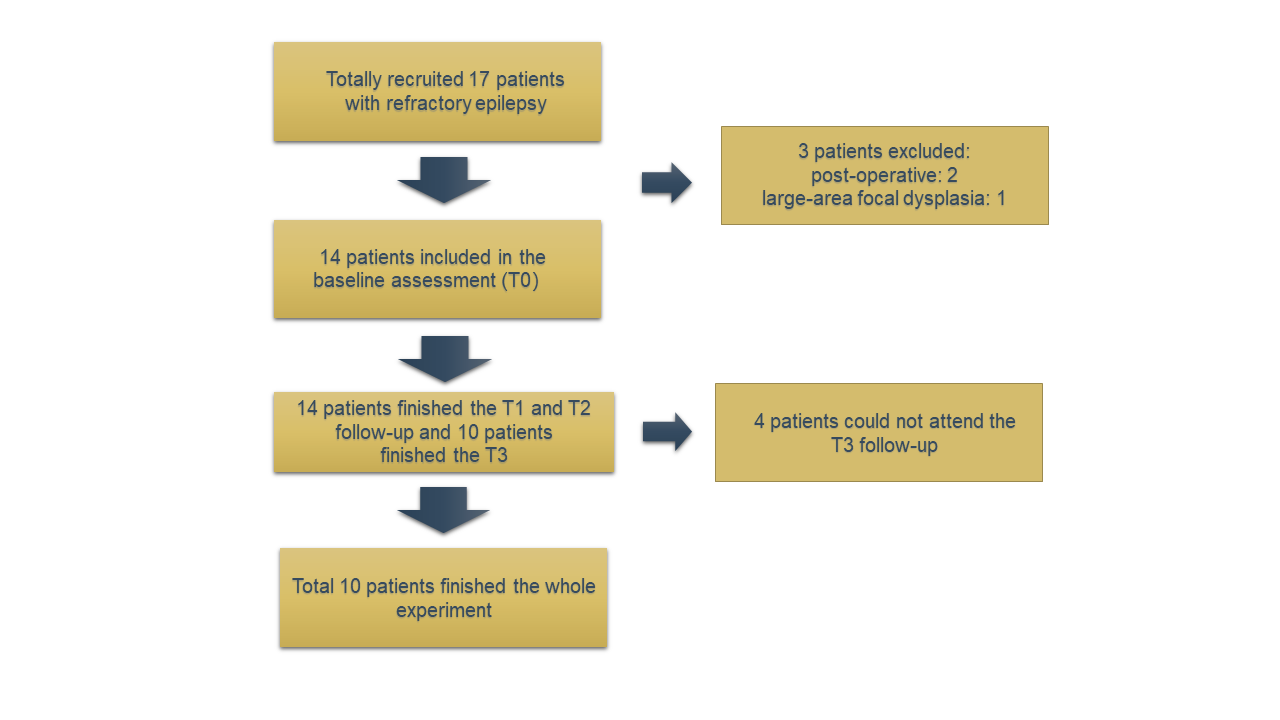
**

**Supplementary Figure 1 Study flow chart of participant.**


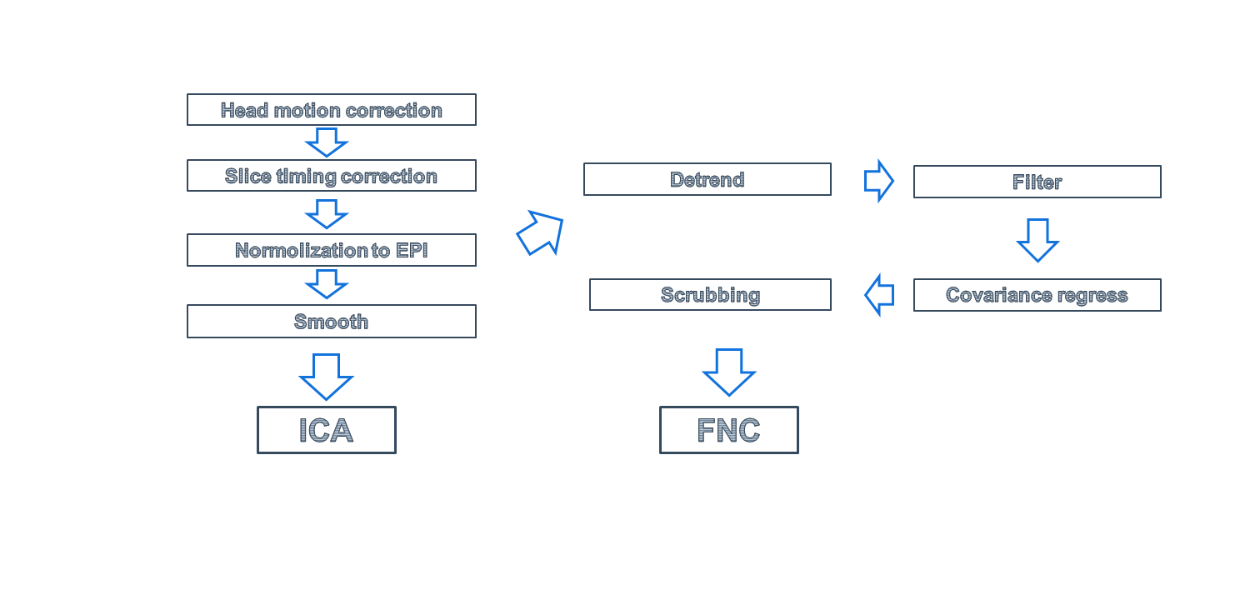


**Supplement Figure 2 Preprocess pipeline.**


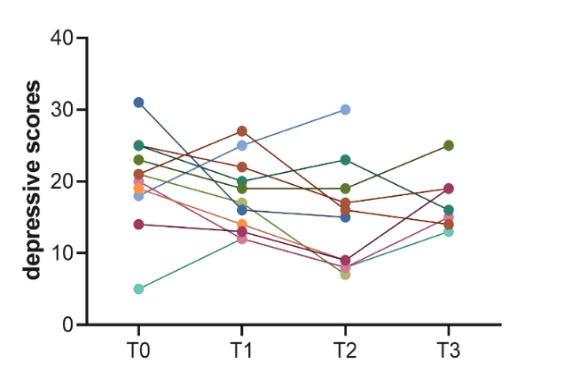

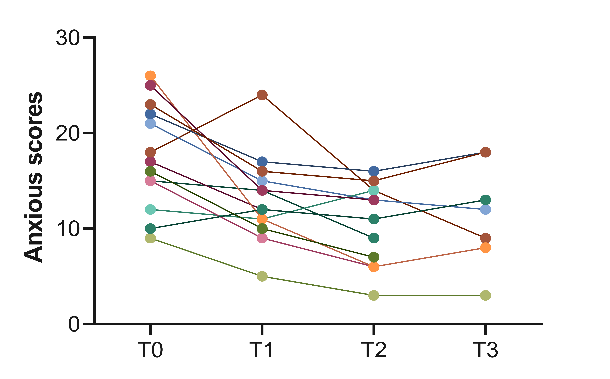

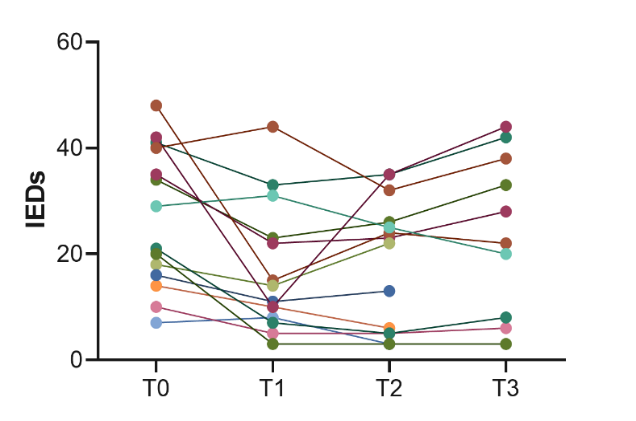

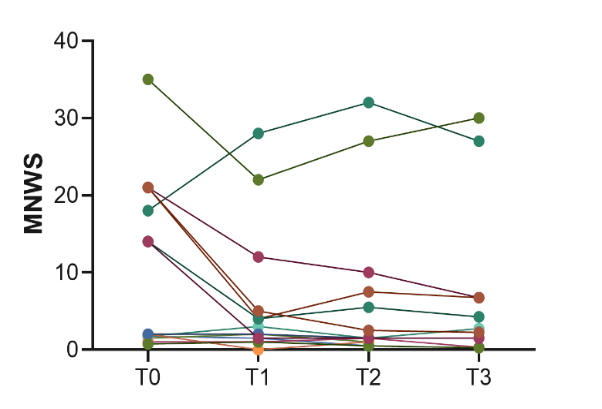


**Supplement Figure 3 Clinical evaluation of rTMS effect for each patient.** Different colors deficted each patients’ clinical indicators, including depressive and anxious scores, interictal epileptogenic discharges(IEDs) and mean number of weekly seizures(MNWS). And four patients withdrew from the T3 evaluation.

Calhoun, V. D., Wager, T. D., Krishnan, A., Rosch, K. S., Seymour, K. E., Nebel, M. B., . . . Kiehl, K. (2017). The impact of T1 versus EPI spatial normalization templates for fMRI data analyses. *Hum Brain Mapp, 38*(11), 5331-5342. doi:<https://doi.org/10.1002/hbm.23737>

Friston, K. J., Williams, S., Howard, R., Frackowiak, R. S., & Turner, R. (1996). Movement-related effects in fMRI time-series. *Magn Reson Med, 35*(3), 346-355. doi:<https://doi.org/10.1002/mrm.1910350312>

Power, J. D., Barnes, K. A., Snyder, A. Z., Schlaggar, B. L., & Petersen, S. E. (2012). Spurious but systematic correlations in functional connectivity MRI networks arise from subject motion. *Neuroimage, 59*(3), 2142-2154. doi:<https://doi.org/10.1016/j.neuroimage.2011.10.018>

Power, J. D., Schlaggar, B. L., & Petersen, S. E. (2015). Recent progress and outstanding issues in motion correction in resting state fMRI. *Neuroimage, 105*, 536-551. doi:<https://doi.org/10.1016/j.neuroimage.2014.10.044>

Wang, J., Wang, X., Xia, M., Liao, X., Evans, A., & He, Y. (2015). GRETNA: A graph theoretical network analysis toolbox for imaging connectomics. *Front Hum Neurosci, 9*, 386. doi:<https://doi.org/10.3389/fnhum.2015.00386>
